# Hepatocellular Carcinoma: Carcinogenesis, Establishment, Progression, and Therapies

**DOI:** 10.1155/2014/706142

**Published:** 2014-06-17

**Authors:** Yong-Song Guan, Mohammad Ahmad Al-Shatouri, Qing He, Wei Mike Liu

**Affiliations:** ^1^Department of Oncology and Radiology, West China Hospital, Sichuan University, Chengdu, Sichuan 610041, China; ^2^Suez Canal Medical School, Suez Canal University, Ismailia 41522, Egypt; ^3^Department of Pediatrics and Simon Cancer Center, Indiana University School of Medicine, Indianapolis, IN 46202-5225, USA

Hepatocellular carcinoma (HCC) has had a continuous increase worldwide in incidence over the last two decades. The development of HCC has a complicated process and correlates to numerous risk factors including hepatitis virus infection, alcoholism, food toxins, diabetes, obesity, smoking, congenital liver diseases, and errors of metabolism. Molecular links are also identified, which characterize tumorigenic intracellular pathways and suggest molecular targeted therapies.

Each year, HCC is diagnosed in approximately 630,000 people worldwide and more than half of the new cases come from China. Correct understanding and interpretation of the details of carcinogenesis, establishment, and progression of this cancer is of paramount importance for its treatment with good outcome. There are many therapeutic modalities for treatment of HCC but evidence-based approach to the multidisciplinary management is substantial.

This special issue contains the research papers focusing on the current general interest in HCC, including basic research, laboratory study, diagnosis, and treatment. Study on molecular aspects of HCC is a highlight of this special issue, involving microRNAs, genes, tumor markers, and so forth. We hope that these articles will stimulate the lasting exertion to unveil the mechanism of HCC development and progression and cast new insights into HCC establishment and management.

*Yong-Song Guan* *Mohammad Ahmad al-Shatouri* *Qing He* *Wei Mike Liu*

